# Adoptive T Cell Therapy for Solid Tumors: Pathway to Personalized Standard of Care

**DOI:** 10.3390/cells10040808

**Published:** 2021-04-05

**Authors:** Shuyang S. Qin, Alexa D. Melucci, Alexander C. Chacon, Peter A. Prieto

**Affiliations:** 1 Department of Microbiology & Immunology, University of Rochester School of Medicine & Dentistry, Rochester, NY 14642, USA; Shuyang_Qin@urmc.rochester.edu; 2 Department of Surgery, University of Rochester Medical Center, Rochester, NY 14642, USA; Alexa_Melucci@urmc.rochester.edu (A.D.M.); Alexander_Chacon@urmc.rochester.edu (A.C.C.)

**Keywords:** adoptive cell therapy, tumor-infiltrating T cells, immunotherapy, metastatic treatment

## Abstract

Adoptive cell therapy (ACT) with tumor-infiltrating T cells (TILs) has emerged as a promising therapy for the treatment of unresectable or metastatic solid tumors. One challenge to finding a universal anticancer treatment is the heterogeneity present between different tumors as a result of genetic instability associated with tumorigenesis. As the epitome of personalized medicine, TIL-ACT bypasses the issue of intertumoral heterogeneity by utilizing the patient’s existing antitumor immune response. Despite being one of the few therapies capable of inducing durable, complete tumor regression, many patients fail to respond. Recent research has focused on increasing therapeutic efficacy by refining various aspects of the TIL protocol, which includes the isolation, ex vivo expansion, and subsequent infusion of tumor specific lymphocytes. This review will explore how the therapy has evolved with time by highlighting various resistance mechanisms to TIL therapy and the novel strategies to overcome them.

## 1. Introduction

**Figure 1:**
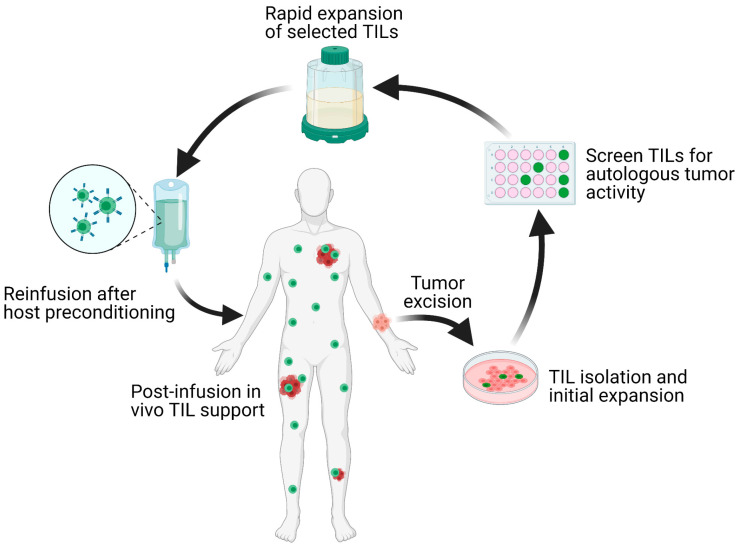
Schematic overview of the TIL-ACT protocol. Patients with metastatic tumors undergo metastastectomy of one lesion, which is then digested into multiple small tumor fragments or single-cell suspensions. Tumor fragments are cultured with IL-2 in vitro for the initial TIL isolation and expansion. Isolated TILs are screened for tumor reactivity via co-culture with autologous digested tumor cells for IFN-γ secretion as assessed by IFN-γ ELISA. Tumor specific TIL clones are then consolidated and rapidly expanded in the presence of anti-CD3 monoclonal antibody, IL-2, and irradiated autologous feeder cells. Once the number of TILs has reached treatment levels (typically > 1 × 10^10^ cells), they are harvested and transferred back into a lymphodepleted host in one infusion. TIL (tumor-infiltrating lymphocytes); ACT (adoptive cell therapy); IL-2 (interleukin-2); IFN-γ (interferon-gamma); ELISA (enzyme-linked immunosorbent assay).

Solid malignancies encompass a wide range of diseases from lymphomas to carcinomas that affect nearly every organ in the body. The malignant transformation from normal cells is accompanied by progressive accumulation of genetic mutations. As a result, patients diagnosed with the same histological tumor type exhibit a wide range of genetic mutations and tumor microenvironments (TME). This inherent heterogeneity in tumors often leads to variations in patient-specific responses. Precision medicine, which refers to the tailoring of treatments to tumor-specific cellular or molecular characteristics, has recently became the mainstay of oncological therapy. The epitome of personalized therapy is adoptive cell transfer (ACT) with autologous tumor-infiltrating lymphocytes (TIL). This process involves tumor excision, isolation of TILs, ex vivo selection for autologous tumor reactivity, rapid expansion, and reinfusion of the T cell product back to the host [1] (Figure 1).

The process of tumorigenesis produces nonsynonymous somatic mutations that lead to the generation of neoantigens. Neoantigens may be derived from (1) translation of mutated genes, (2) aberrant expression of non-mutated genes, and/or (3) the untimely expression of embryonic or cell-lineage specific genes [2,3]. In a similar stochastic process, every T cell expresses a unique T cell receptor (TCR) specific for one antigenic peptide sequence. Recognition of the cognate peptide presented on major histocompatibility complex (MHC) molecules lead to T cell activation and cytolytic target killing [4,5]. This diversity in potential neoantigens and endogenous TCRs poses a significant challenge for the effective immune recognition of tumors. As TIL-ACT specifically utilizes endogenous lymphocytes found within tumors, it increases the probability of enriching for tumor-specific T cells. Other ACT protocols have experimented with the transfer of different lymphocyte populations, including, but not limited to, genetically modified lymphocytes isolated from peripheral blood. In contrast to TILs, which contain endogenous tumor-recognition abilities, these lymphocytes are altered ex vivo to express tumor-specific TCRs and/or to have increased effector functions. [6]. A notable example, which is frequently used to treat hematological malignancies, is the transfer of genetically modified T cells with chimeric antigen receptors (CAR-T) that are specific for previously identified tumor-specific neoantigens [7].

The first evidence that ACT of autologous TILs leads to tumor-specific cytolysis and metastatic tumor control emerged in the 1980 s from murine models of sarcoma, colon adenocarcinoma, melanoma, and bladder carcinoma [8]. In the initial clinical study demonstrating the curative potential of TIL-ACT, 29 metastatic melanoma patients were treated with autologous TILs accompanied by high-dose interleukin-2 (IL-2), with a 31% objective response rate (ORR, complete response (CR) + partial response (PR)), and four patients achieving complete tumor regression [9]. A seminal clinical trial conducted by the Surgery Branch of the National Cancer Institute (NCI) in the early 2000 s demonstrated that the ORR for metastatic melanoma patients can be as high as 72%, with complete response rate (CRR) of 22% following one TIL infusion [10]. Therapeutic response to TIL-ACT is restricted by numerous factors, including, but not limited to, the initial amount of tumor-infiltrating T cells, the presence of immunosuppressive cell types in the TME, and the failure of adoptively transferred cells to persist in the host.

Advances in tumor immunology have led to the gradual optimization of the TIL-ACT protocol from lymphocyte selection to pre-treatment conditioning, and to combination with adjunctive treatment modalities. In this review, we will provide an overview of recent preclinical and clinical advances that have led to the continual improvement of TIL-ACT. We will highlight common mechanisms of resistance as well as novel therapeutic strategies that target these pathways to overcome resistance in the treatment of solid tumor malignancies.

## 2. Mechanisms of ACT-TIL Resistance

**Figure 2:**
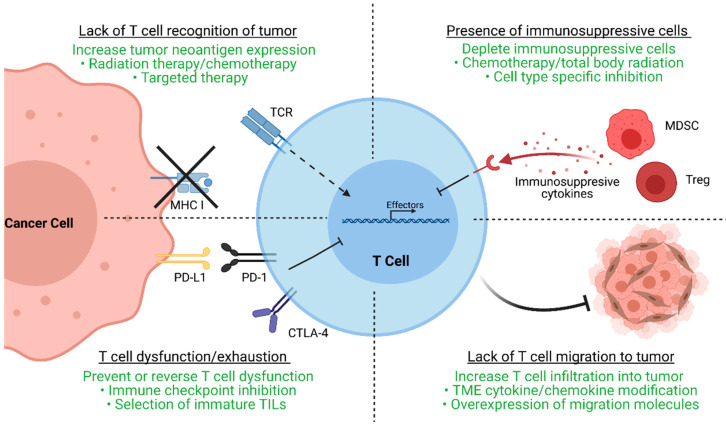
Major resistance mechanisms to TIL-ACT. This figure demonstrates four major resistance mechanisms to TIL therapy as well as strategies (highlighted in green) to overcome these mechanisms. Tumor cells may downregulate MHC molecules or neoantigen expression to avoid immune detection by neoantigen-specific T cells. Radiation therapy, chemotherapy, and targeted therapy are common ways to increase neoantigen generation and upregulate neoantigen presentation. Tumor cells and other components of the TME may upregulate checkpoint molecules such as PD-L1 to induce T cell exhaustion, a dysfunction state by which T cells cannot proliferate or secrete effector molecules. Exhausted T cells can regain effector function with immune checkpoint inhibitor treatment. Another way to bypass T cell exhaustion is by selecting for TILs that are less prone to exhaustion. Immunosuppressive cell types within the TME, such as myeloid-derived suppressor cells (MDSCs) or regulatory T cells (Tregs), can secrete factors that inhibit TIL function. These host immunosuppressive cells can be depleted prior to or simultaneously with adoptive cell transfer by chemotherapy or radiation therapy regimens as well as cell type specific inhibition. Lastly, the TME itself may pose barriers that exclude T cells and inhibit their migration. Experimental therapies that modify chemotactic and migratory pathways may restore or increase tumor infiltration by TILs. TIL (tumor-infiltrating lymphocytes); ACT (adoptive cell therapy); MHC (major histocompatibility complex); TME (tumor microenvironment); PD-L1 (programmed death-ligand 1); PD-1 (programmed cell death protein 1); CTLA-4 (cytotoxic T-lymphocyte-associated protein 4); MDSC (myeloid-derived suppressor cells); Tregs (regulatory T cells).

After the initial clinical trials in metastatic melanoma conducted by the NCI, TIL-ACT has been adopted by numerous other institutions around the world for the treatment of a variety of solid tumors [11,12,13,14]. While objective responses have been replicated in subsequent clinical trials, a significant percentage of patients experience therapy failure by displaying innate or acquired therapy resistance. Innate resistance occurs in patients unresponsive to initial therapy, whereas acquired resistance occurs in patients who were initially responsive but eventually become unresponsive. Regardless of when resistance occurs, the underlying mechanisms result in the lack of generation of functional T cells, or the suppression an otherwise productive T cell response (Figure 2).

As ACT is based on the premise of delivering antitumor T cells to the tumor, the lack of functional, tumor-specific T cells within tumor can abrogate therapeutic efficacy [12]. Effective TILs recognize tumor-specific neoantigens, which are results of stochastic somatic mutation accumulation during tumorigenesis. Thus, cancers with high mutational burdens, such as melanoma and lung cancer, have increased chances of generating neoantigens and are more likely to respond to T cell-based therapies [15,16,17]. In contrast, cancers with lower mutational burden, such as cervical cancer, exhibit decreased response to TIL- ACT (ORR 28%) [18]. However, cytolysis of neoantigen containing tumor clones may lead to the eventual outgrowth of antigen-negative tumor clones that are unrecognizable by TILs. Furthermore, tumor cells may also downregulate MHC molecules or other components of antigen presentation to evade immunosurveillance [19]. Aside from tumor cells, the TME can physically prevent TIL infiltration via generation of dense extracellular matrix, secretion of chemo-repulsive chemokines, or aberrant angiogenesis [20]. The TME can also contain a multitude of immunosuppressive cell types such as myeloid-derived suppressor cells (MDSCs) and regulatory T cells (Tregs). These cells can secrete anti-inflammatory cytokines (IL-10 and TGF-β), deplete essential T cell nutrients from the microenvironment, and suppress antigen presenting cells (APCs) to further inhibit T cell-mediated immunity [21,22]. Ultimately, different TME factors converge to generate a microenvironment conducive to T cell exhaustion, a state in which T cells upregulate checkpoint molecules as they progressively lose the ability to proliferative and perform effector functions [23]. In reality, numerous distinct escape mechanisms contribute to the immunosuppression and therapy resistance of TILs.

Given that every tumor is unique as a result of intra- and inter-tumoral heterogeneity, one must identify and target non-redundant resistance pathways present in each patient to optimize therapeutic benefit. With this knowledge, multiple clinical trials have focused on improving TIL-ACT responses by altering the original protocol based on four main components: (1) ex vivo selection and expansion of tumor-specific T lymphocytes; (2) patient pre-conditioning regimens; (3) post-infusion in vivo TIL support; and (4) potential adjuvant therapies. A partial list of current clinical trials involving TIL-ACT is summarized in Table 1.

## 3. TIL-ACT Protocol Refinement

### 3.1. TIL Cell Type Selection

**Figure 3:**
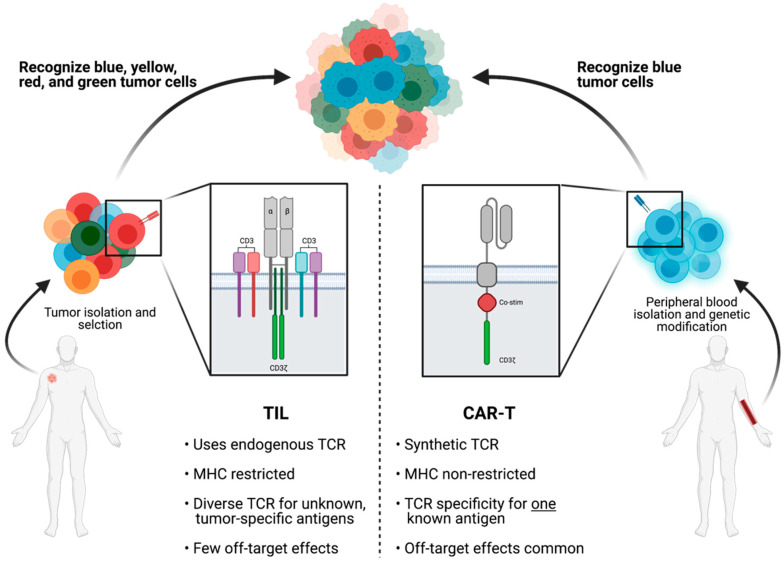
Structural and functional differences between TILs and CAR-Ts in antitumor treatment. ACT can be accomplished using either TILs or CAR-Ts. TILs use endogenous T cell receptors (TCRs) that are MHC restricted. The inherent diversity of TCRs present on TILs allows recognition of a wide range of tumor neoantigens expressed by heterogeneous tumor cells. In contrast, CAR-Ts have synthetic receptors modeled after non-MHC restricted monoclonal antibodies. These receptors contain co-stimulatory and signaling molecules that can independently activate the T cell without recruitment of additional factors. However, as CARs are synthesized to have high affinity binding against one known neoantigen, they are more prone to have off-target effects if the neoantigen is present on normal tissues. TIL (tumor-infiltrating lymphocytes); CAR (chimeric antigen receptor); TCR (T cell receptor); MHC (major histocompatibility complex).

ACT is the process by which endogenous cells are isolated, selected, expanded, and re-infused back into the patient; however, the type of cells transferred ultimately determines the therapeutic effects. Although TILs and genetically modified T cells both derive from autologous lymphocytes and are eventually transferred back to the patient, there exist significant differences between the two (Figure 3). Although a thorough dissection of non-TIL based ACT is beyond the scope for this review, we will briefly compare the use of CAR-T with TIL in the treatment of solid tumors.

TILs typically refer to unmodified T cells with endogenous TCRs, whereas CAR-T cells contain synthetic, modified TCRs. Endogenous TCRs consist of heterodimers that recognize MHC restricted short peptide chains but cannot initiate downstream signaling without the recruitment of additional accessory molecules [24]. In contrast, CAR-T cells express synthetic single-chain receptors derived from monoclonal antibodies that are not MHC restricted and are coupled with T cell signaling domains. First generation CARs consist of a single CD3ζ TCR activation domain, whereas subsequent generations of CARs contain one or more costimulatory molecules to further augment T cell signaling and activation [25,26,27,28]. CAR-T cells can thus automatically activate downstream signaling pathways upon binding to cognate neoantigen without the requirement for engagement of external co-stimulatory molecules or co-receptors. The differences between TCR and CAR dictate the functionality of these T cells in immunotherapy. The diversity of the TCRs allows endogenous TILs to recognize a wider range of unknown tumor neoantigens, while the specificity of the synthetic monoclonal receptor allows high affinity CAR binding to known neoantigens [6,29]. However, this high affinity CAR-T binding to a single neoantigen leads to increased probability of off-target toxicities secondary to antigen presence on normal cells [7]. Whereas the toxicities for ACT-TIL are predominantly attributed to lymphodepletion and IL-2 regimens with rare, minor autoimmunity (vitiligo and uveitis) secondary to TIL infusion, CAR-T infusions have frequently been linked to cytokine release syndrome (CRS) and CAR T-cell-related encephalopathy syndrome (CRES) in addition to toxicities from preparative regimens [7,30]. As a result, CAR-T is preferentially used in the treatment of hematological malignancies with well-characterized mutations, such as CD19 CAR-T for B cell leukemias, while a more diverse set of tumor-reactive TILs is preferred in the ACT of heterogeneous solid tumors [31]. As the generation of CAR-T is limited by the knowledge of receptor sequences targeting specific neoantigens, continued efforts in identifying a wide range of neoantigens unique to solid tumors and subsequent reverse engineering of cognate chimeric receptors is necessary to improve CAR-T cell usage and efficacy.

The generation of a diverse set of tumor-specific TILs is both complex and time-consuming. After initial ex vivo isolation and expansion from a resected tumor, multiple T cell clones are selected against autologous tumor cell lines for IFN-γ activity, which acts as a surrogate marker for tumor-reactivity. Clones negative for tumor-specificity are eliminated while the rest undergo rapid expansion to generate the ultimate infusion product which typically contains at minimum 1 × 10^10^ cells [32]. The entire process from tumor resection to cell administration normally takes 6–8 weeks [33]. However, objective clinical response to TIL-ACT has been associated with increased average telomere length in transferred TILs (6.7 kb in CR, 6.2 kb in PR vs. 5.1 kb in no response (NR), *p* = 0.006), which is inversely associated with the ex vivo culture time [14,34]. To reduce culturing time, the Surgery Branch at the NCI subsequently developed a TIL-ACT protocol with “young” TILs that are enriched for CD8+ T cell but not additionally selected for tumor reactivity. Patients treated with “young” TILs exhibited similar ORR as those treated with traditional TILs. However, as the new protocol accelerated and increased the success rate for TIL generation, it has since been adopted by other institutions [13,35,36].

While other short-term selection methods have been attempted, inherent intra- and intertumoral heterogeneity complicates the selection process. The NCI has recently developed an unbiased high-throughput TIL screen against autologous neoantigens to circumvent this issue. In this novel protocol, TILs are selected against autologous antigen presenting dendritic cells (DC) pulsed with peptide pools or tandem minigenes derived from nonsynonymous mutations identified by whole-exome sequencing of the tumor [37,38,39]. This highly sensitive screen requires less time than traditional autologous tumor cell co-cultures. Furthermore, it is capable of isolating multiple tumor-specific T cell clones when the traditional screen failed in a patient that otherwise would have been excluded from receiving TIL-ACT [31,40]. However, as whole exome sequencing remains costly, the widespread usage of this selection method is currently limited.

Multiple clinical trials have associated objective treatment responses to increasing total number of infused TIL, and more specifically of CD8+ T cells [14,35,41,42]. Tumors isolated from patients with low lymphocytic infiltration often fail to generate sufficient numbers of T cells required for re-infusion (median of 8% lymphocytic infiltration for failed cultures vs. ~50% successful cultures, *p* = 5 × 10^−8^) [36]. In contrast, no correlation has been identified between the number of CD4+ T cells and treatment response. The heterogeneity within overall CD4+ T cell population may contribute to its ambiguous role in antitumor immunity. CD4+ T cells can be characterized into helper T cells (T_H_1, T_H_2, and T_H_17) and Tregs. Tregs secrete anti-inflammatory cytokines that suppress ongoing immune responses, and their presence is associated with poor clinical prognosis [43,44]. In contrast, helper T cells secrete pro-inflammatory cytokines and chemokines that enhance antitumor responses and mediate tumor regression in preclinical models [44,45,46]. Case reports have shown the potential of adoptively transferred tumor-infiltrating T_H_1 and of bulk CD4+ T cells in mediating transient tumor regression in cholangiocarcinoma and melanoma, respectively, via tumor antigen-specific secretion of IFN-γ [47,48]. However, the adoptive transfer of CD8+ T cell enriched TIL products containing minimal CD4+ T cells results in ORRs similar to that of bulk TILs, indicating that CD4+ T cells do not significantly contribute to the observed therapeutic response [35,49].

Another characteristic that has been positively correlated with objective treatment response is the persistent survival of transferred, functional TILs in the patient [10,11,42,50]. Tumor-specific TILs have been detected within peripheral blood of responsive patients for up to 34 months post infusion [11]. Furthermore, in one clinical study, increasing response to therapy has been correlated with increasing half-lives of tumor-specific TIL clonotypes (132–173 days for CR vs. 31–53 days for PR and 13–15 days for NR, *p* < 0.05) [51]. Often, in vivo persistence and clinical response are dependent on the type of TIL transferred. In fact, effector CD8+ T cells derived from less differentiated precursors, such as central memory and naïve T cells, demonstrate increased secretion of effector molecules and proliferation over those derived from the heterogeneous bulk TIL populations [52]. Conversely, the transfer of more differentiated T cells leads to impaired antitumor efficacy and decreased overall survival in preclinical models [53,54]. Single cell analysis of different TIL clonotypes in a patient with metastatic colorectal cancer exhibiting partial response revealed a set of genetic signatures associated with T cell persistence that resembles less differentiated T cells. Of note, compared to non-persistent cells, persistent cells express decreased levels of *EOMES* (transcription factor upregulated in terminally exhausted T cells), and elevated levels of *IL7R* (receptor for homeostatic proliferation cytokine IL-7) [55,56,57]. Thus, the selection for CD8+ T cells with more progenitor-like phenotypes may increase the ORR of TIL-ACT.

### 3.2. Role of Cytokine Support

Cytokines play an indispensable role in the generation, activation, and proliferation of lymphocytes. As objective responses to TIL-ACT have been linked to the continual persistence of adoptively transferred lymphocytes in vivo, the cytokines used for the ex vivo expansion and post-infusion support of TIL become important determinants of treatment efficacy.

IL-2, IL-7, IL-12, and IL-15 all affect T cell differentiation and, thus, are optimal candidates for the ex vivo expansion and differentiation of TILs used for therapy. IL-2 is a pleiotropic cytokine that induces cell-type specific responses. For example, IL-2 is not only involved in the differentiation and homeostasis of Tregs but it also promotes the differentiation of effector CD8+ T cells and increased synthesis of effector molecules [44,58,59]. Since the initial clinical trials in the 1980s, TIL-ACT protocols have incorporated the use of IL-2 in the ex vivo expansion phase [9,32]. Thus far, this has led to a highly reproducible and largely successful generation of sufficient numbers of TIL for subsequent adoptive transfer. With increasing understanding of how different lymphocytic subsets mediate tumor regression, other cytokines are currently being considered to enrich for more effective T cells. IL-12 is a potential antitumor cytokine that can both directly augment T cell cytolytic potential and increase antigen presentation [60]. Murine models indicate that IL-12 mediated activation of naïve T cells generate highly proliferative progenies with elevated cytolytic function and increased resistance to exhaustion [61,62,63]. Furthermore, the adoptive transfer of IL-2 + IL-12 primed T cells lead to enhanced tumor regression in multiple studies [61,62,64]. In contrast, IL-7 and IL-15 have been identified as critical factors for maintaining in vivo homeostatic T cell proliferation and function, particularly of memory T cells [57,65,66]. However, the combination of IL-7 + IL-15 does not result in superior ex vivo generation of effector T cell for ACT than when compared to IL-2 [67].

Beyond ex vivo expansion, TIL protocols also use high-dose (HD) IL-2 as an adjuvant to support lymphocyte proliferation in vivo after transfer [32,68]. However, systemic IL-2 treatment is associated with severe toxicities that restrict its widespread clinical usage [14,69,70]. In fact, most patients do not complete the entire post-transfer IL-2 regimen due to overwhelming adverse reactions, including, but not limited to, vascular leak syndromes and neurological symptoms [71]. Many clinical trials have explored the use of attenuated or low-dose (LD) IL-2. This dosage reduction leads to better tolerance of the therapy and less severe, more transient adverse events [72,73,74,75]. A meta-analysis of the efficacy of HD (*n* = 332) vs. LD IL-2 (*n* = 78) with TIL-ACT showed that the pooled ORR (CRR) was estimated to be 43% (14%) and 35% (7%), respectively [71]. Hence, LD IL-2 regimens have the potential to elicit durable responses while minimizing associated adverse effects. However, at this point, clinical trials involving LD IL-2 have limited patient enrollment with no trial directly comparing the efficacy of HD vs. LD IL-2 as adjuvants to TIL-ACT.

### 3.3. Role of Pre-Conditioning Regimens

The necessity of modifying the host immune environment prior to TIL transfer has been long established as therapeutic effects were only achieved with the addition of cyclophosphamide [8,12,76]. Currently, the main preparative conditioning regimens for TIL-ACT consist of non-myeloablative chemotherapy (NMA) with cyclophosphamide + fludarabine and/or total body irradiation (TBI). Both methods result in the depletion of host lymphocyte-mediated immunosuppressive factors that potentially hinder the functions of transferred T cells [77,78]. Further research indicates that lymphodepletion can eliminate homeostatic cytokine sinks, inhibit immunosuppressive Tregs, and increase neoantigen presentation [53,79,80]. The eradication of endogenous cells that compete for homeostatic cytokines IL-7 and IL-15 leads to an increase in serum levels that is thought to be beneficial for TIL persistence [42,69,81]. The removal of host tumor-infiltrating and peripheral CD4+ CD25+ FoxP3+ Tregs liberates adoptively transferred TILs from extrinsic immunosuppression [43,44,82]. Lastly, preconditioning may induce tumor cells to undergo immunogenic cell death, resulting in the release of pro-inflammatory cytokines and tumor neoantigens that further augments the antitumor immune response [83].

The association of increased lymphodepletion intensity to increasing TIL-ACT efficacy further demonstrates the importance of preparative regimens [84]. In a clinical trial conducted by the NCI, the addition of 2 or 12 Gy irradiation to preconditioning NMA chemotherapy increased the ORR from 49% (21/43 patients) to 52% (13/25 patients) and 72% (18/25 patients), respectively, in metastatic melanoma patients treated with TIL-ACT [69]. Subsequently, a randomized trial of NMA chemotherapy vs. NMA + 12 Gy TBI showed similar complete response rates (24% in both), but latter group had more partial responders (22% vs. 38%) [42]. While patients typically recover endogenous bone marrow function accompanied by a return of peripheral hematological values to normal limits within 2 to 3 weeks after lymphodepletion, the majority does experience severe treatment-related toxicities requiring advanced medical support [69,85,86]. Thus, the optimization of preconditioning regimens that minimize adverse events is necessary to increase the clinical utility of TIL-ACT.

## 4. Combination Treatments

### 4.1. Immune Checkpoint Inhibitors

A common immunotherapy resistance mechanism exhibited by various types of tumors is the upregulation of negative T cell checkpoint pathways, such as programmed cell death protein 1 (PD-1) and cytotoxic T-lymphocyte-associated protein 4 (CTLA-4), in the TME. Chronic co-inhibitory signaling induces T cell exhaustion, a functional state defined by the loss of effector functions and proliferative capability [87,88,89]. Accumulation of exhausted T cells eventually lead to tumor progression and immune evasion. Without extensive TME changes, any adoptively transferred TILs would be subjected to the same external suppression mechanisms, nullifying the therapy. However, blocking these pathways with immune checkpoint inhibitors (ICIs) can re-invigorate previously exhausted T cells to promote metastatic tumor regression [90]. Recently, ICIs against PD-1 and CTLA-4 have been approved by the Food and Drug Administration (FDA) for the treatment of numerous solid malignancies [91].

Although the characterization of TIL products demonstrate increased PD-1 expression on tumor-reactive T cells [11,92,93,94], chronic persistence of PD-1 expression on post-transfer T cells is associated with therapeutic resistance [72]. The existence of PD-1+ TIL cells may indicate potential sensitivity to ICI. Preclinical data suggest that the combination of ICI with TIL-ACT can synergistically increase T cell cytolytic ability, effector molecule synthesis, and tumor infiltration, leading to greater tumor control and improved survival [95,96,97,98]. In one case study, combinatory treatment of TIL and a PD-1 inhibitor led to complete regression of all six metastases in a patient with metastatic breast cancer [99]. Furthermore, multiple metastatic melanoma patients who previously failed ICI treatments were able to achieve partial response with TIL and went on to have durable complete responses with subsequent ICI treatment [14,75]. Patients treated with ACT of peripheral blood-derived, antigen-specific T cells in combination with CTLA-4 blockade also achieved complete response after prior progression on salvage CTLA-4 therapy [100]. These results suggest the possibility of ICI potentiating adoptively transferred TIL to elicit complete tumor regressions in vivo. A small pilot trial for the combination of TIL-ACT with nivolumab (anti-PD-1) in ovarian cancer demonstrated the safety and feasibility of this combination with two out of six patients achieving objective response [101]. Other on-going clinical trials aiming to assess the efficacy of TIL-ACT and ICI in solid tumors are listed in Table 1**.**

### 4.2. Targeted Therapy

The advent of genetic sequencing has led to the identification of common driver mutations in solid tumor malignancies. Targeted therapies are designed to specifically kill tumor cells expressing these oncogenes. Increased tumor lysis generates a more pro-inflammatory TME as well as releases additional tumor neoantigens to be recognized by TILs. Although preliminary experiments exploring targeted therapy combined with TIL-ACT have only been conducted in melanoma at this point, there exists potential for the extrapolation of this combination in the treatment of other solid tumors with identifiable driver mutations.

Roughly 50% of melanomas carry the BRAF^V600E^ or BRAF^V600K^ driver mutations that are targetable by BRAF inhibitors (BRAFi) such as vemurafenib and dabrafenib [102]. BRAFi mediated apoptosis of BRAF-mutant cells results in transient tumor regression [103]. Preclinical studies suggest that the addition of BRAF inhibition to TIL-ACT can increase T cell tumor infiltration, induce antigen presentation, and sensitize BRAF mutant melanoma to T cell mediated cytolysis [104,105,106,107]. A clinical trial investigated the combined efficacy of BRAFi with TIL-ACT where patients with BRAF^V600^ mutations underwent metastastectomy for TIL growth, followed by two weeks of vemurafenib, another metastastectomy, standard TIL infusion, and resumption of vemurafenib for up to two years. Patients on combination therapy demonstrated an ORR of 64% (*n* = 11 patients), which was similar to the 60% ORR (*n* = 15 patients) of TIL-ACT monotherapy [108]. However, metastases isolated from patients following combination treatment do exhibit increased T cell infiltration compared to monotherapy, suggesting the existence of potential synergistic effects of BRAFi pretreatment with TIL-ACT.

Another targeted therapy commonly used in the treatment of malignant melanoma is MEK inhibitors (MEKi). MEKi are typically added to BRAFi to prevent acquired resistance and patients on this combination experience drastic but transient tumor regression, with median progression-free survival of less than 10 months [109]. The mechanism by which MEKi affects the T cell mediated immunity is relatively unknown due to contradictory preclinical data. While some murine studies demonstrated that neoadjuvant MEK inhibition generate memory T cells with increased effector functions, others have shown that MEKi impairs T cell proliferation and function [110]. Given the lack of concrete evidence, further investigation is needed to determine whether MEKi offers additional clinical benefits when used in combination with TIL-ACT or BRAFi + TIL-ACT.

### 4.3. Other Investigative Therapies

Other adjuvant therapies aimed at (1) increasing tumor infiltration of TILs, (2) enhancing T cell antitumor potentials, or (3) alleviating TME immunosuppression have been explored in both preclinical and small clinical settings. Some strategies for increasing tumor infiltration of adoptively transferred TIL include, but are not limited to, overexpression of adhesion molecule CD62L or chemokine receptor CXCR2 on TILs [95,111], along with intratumoral overexpression of chemokine CCL21 [112]. Alternatively, cytokines such as IL-12 and tumor antigen-specific vaccinations have also been shown to augment transferred TIL effector function and prolong their survival [113,114,115,116]. In a proof-of-concept clinical trial, patients with tumor neoantigen NY-ESO-1 positive advanced sarcoma or melanoma were treated with ACT of NY-ESO-1 specific T cells in combination with NY-ESO-1 DC vaccination. Two out of six patients experienced objective response, with one attaining complete tumor regression [117]. This study suggests the potential of combining tumor vaccination with ACT.

Recent advances in identifying immunosuppressive MDSCs within the TME indicate potential for modifying the immune microenvironment to augment TIL efficacy. Additional activation of antigen presenting cells and macrophages by agonist CD40 monoclonal antibodies leads to enhanced T cell antitumor activity and proliferation [118,119]. Inhibition of MDSC migration by CXCR1/2 inhibitors resulted in increased accumulation of adoptively transferred TILs in murine models of melanoma, lung, and oral carcinomas [120]. Furthermore, MDSC depletion via doxorubicin and docetaxel in combination with TIL-ACT increased T cell tumor-infiltration and enhanced tumor control in preclinical models of melanoma and breast cancer [121,122]. However, due to the preliminary nature of these studies, further investigation is necessary to determine if these strategies could provide additional benefits to TIL-ACT and be safely translated into the clinical setting.

## 5. The Future of TIL-ACT

**Figure 4:**
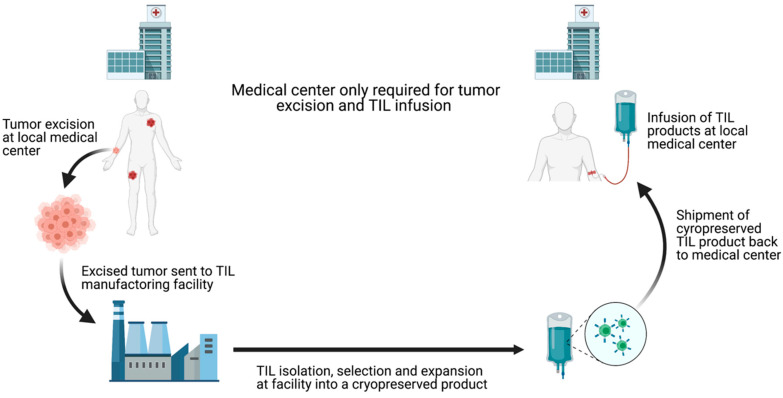
Schematic depiction of centralized TIL production chain. Commercialization of TIL production can increase TIL-ACT implantation by reducing the requirements for local medical centers. By outsourcing TIL production, treatment facilities only need to have the surgical and medical capacities to remove the tumor and treat potential infusion-associated adverse effects. TIL (tumor-infiltrating lymphocytes); ACT (adoptive cell therapy).

Although the clinical success of TIL-ACT has been replicated across multiple institutions, its availability has so far been limited to large academic medical centers. These centers have the expertise to successfully grow TILs and the capability to adequately manage adverse events patients experience [123]. The ex vivo TIL expansion protocol is labor-intensive and the requirement to use fresh TIL products for infusion impose further restrictions on the implantation. Outsourcing TIL production to dedicated manufacturing centers is necessary to increase the cost-effectiveness and expand the availability of this therapy [124]. Iovance Biotherapeutics has established a centralized, high-throughput manufacturing facility for the production of cryopreserved TILs (brand named Lifleucel). Iovance’s model involve receiving surgically resected tumors shipped by different medical centers, expanding TILs to therapeutic numbers in their central facility, and shipping cryopreserved TIL products back to the medical centers [125] (Figure 4). This alleviates the burdens on medical centers and theoretically allows TIL-ACT to be administered anywhere that has the capacity to surgically excise tumors and medically monitor patients during the reinfusion. Furthermore, cryopreserved TIL products offer more flexibility in scheduling as opposed to the rigid timeline dictated by the use fresh TILs. Despite the fact that TIL-ACT has yet to be FDA approved for the treatment of solid malignancies, strong preclinical and clinical results support its potential in eliciting durable tumor regression.

## 6. Conclusions

Preclinical studies and early phase clinical trials of TIL-ACT have shown promising results in the treatment of solid tumors, including the potential for eliciting durable complete tumor regression. TIL-ACT takes advantage of the body’s own immune system and intrinsic antitumor immunity. Autologous TILs are isolated, expanded, and selected for tumor reactivity in this epitome of personalized medicine. Despite the intimate nature of the therapy, a significant amount of patients develop resistance and fail to objectively respond to TIL-ACT. With increasing knowledge of tumor immunology, TIL research has been focused on refining all parts of TIL-ACT protocol to overcome various resistance mechanisms and improve overall therapeutic efficacy. From the initial trial conducted by Rosenberg et al. in the 1980s, the ORR for unresectable melanoma patients treated with TIL-ACT has improved from 31% to as much as 72%, and its usage has been extended to the treatment of other solid tumor [10,32]. The addition of other adjuvant therapies, including, but not limited to, targeted therapy, immune checkpoint inhibitors, monoclonal antibody therapy, and other biologics, may further enhance TIL efficacy via non-redundant pathways and prevent subsequent tumor immune escape. Continual protocol refinement and larger clinical trials of these new avenues can facilitate the widespread adaptation of this personalized immunotherapy for the treatment of malignant solid tumors.

## Figures and Tables

**Table 1:** List of current and recent clinical trials involving TIL-ACT. Clinical trials are separated into sections dependent on the primary study goal. Derivation from the standard protocol (TIL with cyclophosphamide + fludarabine preconditioning and high dose IL-2 post-conditioning) or experimental therapies are bolded.

Reference Number	Cell Type	Pre- and Post-Conditioning	Adjuvant Treatment	Tumor Types	Site Location	Status
TIL-ACT EFFICACY TRIALS						
NCT03778814	TIL enriched for tumor specificity	N/A	N/A	Solid tumors NSCLC	Second Affiliated Hospital of Guangzhou, Medical University, China	Recruiting 12/2021
NCT04596033	TIL	IL-2 +/− Cyclophosphamide Fludarabine	N/A	Multiple advanced solid tumors	Genocea Biosciences, Inc., Nashville, TN	Recruiting 5/2024
NCT04625205	TIL	N/A	N/A	Advanced and metastatic melanoma	BioNTech US Inc., Amsterdam, Netherlands	Recruiting 11/2023
NCT03610490	TIL	Cyclophosphamide Fludarabine IL-2	N/A	Refractory and metastatic ovarian, PDAC, and CRC	M.D. Anderson Cancer Center Houston, TX	Active 09/2021
NCT03449108	Cryopreserved TIL	Cyclophosphamide Fludarabine IL-2	N/A	Bone sarcoma Sarcoma Thyroid	M.D. Anderson Cancer Center Houston, TX	Recruiting 12/2022
NCT03935893	TIL	Cyclophosphamide Fludarabine IL-2	N/A	Multiple solid advanced cancers	University of Pittsburgh Medical Center, Pittsburgh, PA	Recruiting 6/2030
NCT03658785	TIL	Cyclophosphamide Fludarabine IL-2	N/A	Recurrent, metastatic, persistent carcinoma not amenable to currenttreatments	Tongji Hospital China	Not yet recruiting 12/2024
NCT03991741	T cell	IL-2	N/A	Locally advanced and metastatic melanoma, Locally advanced and metastatic head and neck cancer	Immunotherapy Foundation, San Diego, CA	Recruiting 08/2023
PRECONDITIONING						
NCT03992326	TIL	Cyclophosphamide Fludarabine +/− **IL-2** **LDI**		Breast cancer NSCLC Colon cancer Ovarian cancer Other solid tumors (excluding brain, cutaneous, mucosal, ocular/uveal)	Centre Hospitalier, Universitaire Vaudois Lausanne, Switzerland	Recruiting 09/2025
NCT04643574	TIL enriched for tumor specificity	Cyclophosphamide Fludarabine +/− **IL-2** **LDI**		Solid tumors except CNS	Centre Hospitalier, Universitaire Vaudois Lausanne, Switzerland	Not yet recruiting 11/2027
			IL-2 DOSAGE			
NCT01462903	TIL	**Low dose IL-2**	N/A	Hepatocellular carcinoma Breast carcinoma Nasopharyngeal carcinoma	Sun Yat-sen University, China	Unknown
ADJUVANT THERAPIES						
NCT00002733	TIL	IL-2	**Cimetidine** **IFN-α**	Metastatic melanoma Metastatic RCC	Hoag Memorial Hospital Presbyterian, Newport Beach, CA	Completed 01/2000
NCT02876510	TIL	Cyclophosphamide Fludarabine IL-2	**Atezolizumab**	Advanced solid cancers	Immatics US, Inc., Houston, TX	Active not recruiting 12/2021
NCT03645928	Cryopreserved TIL	Cyclophosphamide Fludarabine IL-2	**Pembrolizumab** **Ipilimumab** **Nivolumab**	Metastatic melanoma SCC of the head and neck NSCLC	Iovance Biotherapeutics, Inc., San Carlos, CA	Recruiting 12/2024

TIL (tumor-infiltrating lymphocytes); ACT (adoptive cell therapy); N/A (not applicable); IL-2 (interleukin-2); LDI (low dose irradiation); CNS (central nervous system); NSCLC (non-small cell lung cancer); SCC (squamous cell carcinoma); HCC (hepatocellular carcinoma); RCC (renal cell carcinoma); IFN-α (interferon alpha); PDAC (pancreatic ductal adenocarcinoma); CRC (colorectal cancer).

## Data Availability

Data sharing not applicable.
